# Correlation of Body Mass Index and Serum Parameters With Ultrasonographic Grade of Fatty Change in Non-alcoholic Fatty Liver Disease

**DOI:** 10.5812/ircmj.12669

**Published:** 2014-01-05

**Authors:** Ghobad Abangah, Atefeh Yousefi, Rouhangiz Asadollahi, Yousef Veisani, Paria Rahimifar, Sajjad Alizadeh

**Affiliations:** 1 Department of Gastroenterology and Hepatology, Ilam University of Medical Sciences, Ilam, IR Iran; 2 Student Research Committee, Ilam University of Medical Sciences, Ilam, IR Iran; 3 Department of Pathology, Ilam University of Medical Sciences, Ilam, IR Iran; 4 Department of Clinical Epidemiology, Ilam University of Medical Sciences, Ilam, IR Iran

**Keywords:** Non-alcoholic Fatty Liver Disease, Ultrasonography, Body Mass Index, Aspartate Aminotransferase, Triglyceride

## Abstract

**Background:**

Non-alcoholic fatty liver disease (NAFLD) is a common liver disease in the western population and expanding disease in the world. Pathological changes in fatty liver are like alcohol liver damage, which can lead to end-stage liver disease. The prevalence of NAFLD in obese or overweight people is higher than general population, and it seems that people with high Body Mass Index (BMI) or abnormality in some laboratory tests are more susceptible for severe fatty liver and high grade of NAFLD in ultrasonography (U.S).

**Objectives:**

This study aimed to evaluate the correlation of BMI and laboratory tests with NAFLD in ultrasonography.

**Materials and Methods:**

During a multi-step process, we selected two-hundred and thirteen cases from four hundred and eighteen patients with NAFLD. Laboratory tests performed included: ALT, AST, FBS, Triglyceride and cholesterol levels, hepatitis B surface antigen, hepatitis C antibody, ceruloplasmin, serum iron, TIBC, transferrin saturation, ferritin, AMA, ANA, ANTI LKM1, serum protein electrophoresis, TSH, anti TTG (IgA). BMI and ultrasonography for 213 patients were performed, and then data was analyzed. These parameters and grades of ultrasonography were compared with the values obtained using one way ANOVA. An ordinal logistic regression model was used to estimate the probability of ultrasonography grade. The Statistical Package for the Social Science program (SPSS, version 16.0) was used for data analysis.

**Results:**

Two-hundred and thirteen cases including 140 male and 73 female, were studied. In general, 72.3% of patients were overweight and obese. Post-hoc tests showed that only BMI (P < 0.001) and TG (P < 0.011) among variables had statistically significant associations with ultrasonography grade (USG), and ordinal logistic regression model showed that BMI and AST were the best predictors.

**Discussion:**

Our results suggest that in patients with NAFLD, BMI and TG are most effective factors in severity of fatty liver disease and ultrasonography grade (USG). On the other hand, BMI as a predictor can be helpful. But, AST has not been a reliable finding, because it changes in many conditions.

## 1. Background

Non-alcoholic fatty liver disease (NAFLD) is a common liver disease in the west and an expanding disease in the world (1-4). NAFLD means accumulation of ample fat (5%–10% of organ weight) in the liver in a person who consumes no more than 30 gr alcohol per day in men and 20 gr in women (4-6). In the pathological view of NAFLD, a liver damage is similar to alcoholic-induced liver injury. These patients show a wide range of signs and symptoms. It can be asymptomatic or lead to end-stage liver disease (7, 8). The prevalence of fatty liver in Iran general population is 2% (9, 10). In addition, among obese people is far higher than general population (11, 12). Some studies have proposed that obese or overweight people have more advanced NAFLD (13, 14).

Diagnostic methods for NAFLD are various and include using laboratory tests with imaging methods or liver biopsy (5, 15). Some tests include: Alanine aminotransferase (ALT) and aspartate aminotransferase (AST), Triglyceride (TG), Hepatitis B or C serologies, autoimmune and Wilson's disease (9, 14). Among imaging methods, Magnetic Resonance Imaging (MRI) is the gold standard for diagnose of fatty liver, but the usage of MRI is limited because it is expensive (16-18). Also we can use computed tomography (CT) scan and ultrasonography (U.S) (17). Altogether , except some liver changes like focal and patchy, U.S is more sensitive than CT scan to detect fatty change in liver (18). U.S is a useful, safe and non-invasive method which provides appropriate information about hepatic steatosis (H.S) (19, 20).

Commonly, NAFLD is diagnosed by U.S and divided into mild, moderate and severe stages. In mild stage 10-30 %, moderate 30-70 %, and severe more than 70% of the hepatocytes are involved (18). High grade of NAFLD in U.S is related to the end stage of liver disease (21, 22). However, U.S is not sensitive towards detection of liver inflammation, so biopsy of liver is required to confirm the inflammation, degree of H.S and prognostic information (5, 15, 19, 23, 24). Some factors that seem effective in the process and progresses of NAFLD and ultrasonography grade (USG) include: obesity and Body Mass Index (BMI), diabetes, hyperlipidemia, age and metabolic syndrome (MS) (25-27). However, there is controversy about the effect of liver enzymes or lipid profile. Some articles agreed to the effect of liver enzymes on U.S grades and others indicated that only lipid profile or some liver enzymes could affect the process of disease and the grade of U.S (26-30).

## 2. Objectives

In this article, we investigated the associations between serum parameters and Body Mass Index changes with the ultrasonography grade in patients with NAFLD.

## 3. Materials and Methods

### 3.1. Study Designs

It was a cross-sectional study.

### 3.2. Study Population

**Figure 1.**
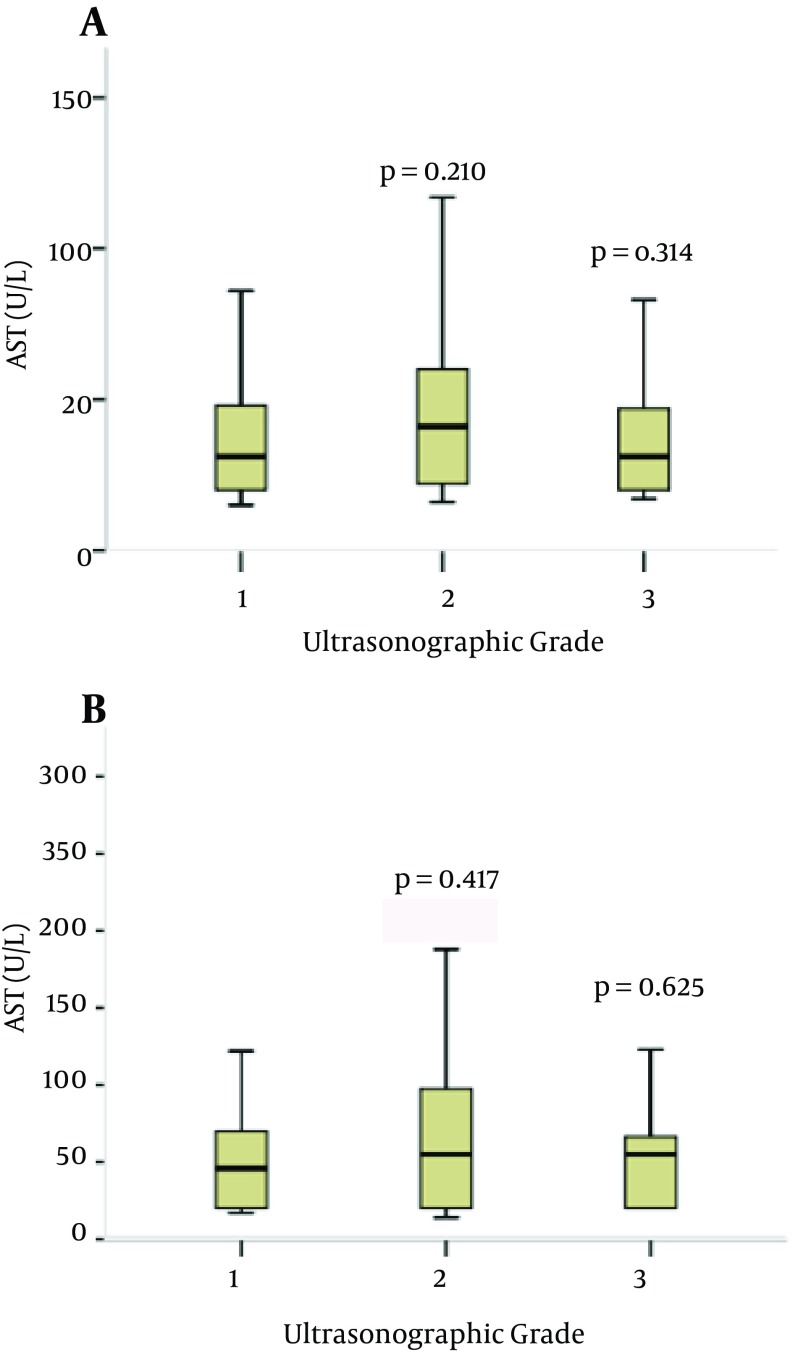
Serum Alanine Aminotransferase (ALT) and Aspartate Aminotransferase (AST) Levels in Different Ultrasonographic Groups. P values are for the Analysis of Variance (Post-hoc Comparisons) Considering the Mild Ultrasonographic Group as the Reference Category

All patients with clinical and evidence of NAFLD were selected from January to November 2012 at the Gastroenterology and Hepatology Clinic, Ilam University of Medical Sciences, Ilam, Iran (Step 1). Four hundred and eighteen patients selected during Step 1. Inclusion criteria were: patients without alcohol use or occasional use (< 30gr alcohol per day in men, and < 20gr in women). Exclusion criteria were: chronic hepatic disease (hepatitis B and C, hemochromatosis), systemic comorbidities and neoplasm, hepatotoxic drugs during the past 6 months (Step 2) (14, 31). At first, we checked hepatitis B surface antigen, hepatitis B core antigen, hepatitis C antibody, and ceruloplasmin. Next, we checked AST, ALT, Fasting Blood Glucose (FBS), cholesterol, TG and USG of the liver for 213 patients recruited in this study (Step 3) (Figure 1). 

### 3.3. Study Measurements

The height (cm) and weight (kg) and Body mass index of cases were measured according to the World Health Organization (WHO) criteria (32) Patients' weight was measured with light clothes, without shoes (by Seca sensa 804, Hamburg, Germany), and then height was measured (by Seca 206, Hamburg, Germany). Body mass index (BMI; kg/m^2^) was calculated for all subjects by dividing a person's weight in kilograms by the square of their height in meters. Patients were classified as normal weight (BMI < 25.0 kg/m^2^), overweight (BMI ≥ 25.0 and ≤ 29.9 kg /m^2^), and obese (BMI ≥ 30.0 kg /m^2^).

### 3.4. Laboratory Investigations

To evaluate the autoimmune hepatitis, hemochromatosis and other exclusion criteria we checked: hepatitis B surface antigen, hepatitis C antibody, ceruloplasmin, Serum Iron (SI), Total iron-binding capacity (TIBC), transferrin saturation (TS), ferritin, Anti Mitochondrial Antibody (AMA), Anti-Nuclear Antibody (ANA), ANTI LKM1, serum protein electrophoresis, Thyroid-stimulating hormone (TSH), and anti TTG (IgA). liver enzymes as alanine aminotransferase (U/L) (ALT), aspartate aminotransferase (U/L) (AST) and Fasting Blood Glucose (mg/dL) (FBS), triglyceride (mg/dL) (TG) and cholesterol levels (mg/dL) were determined by using Auto Analyzer Alpha Classic (Tehran, Iran) and Pars Azmoon reagent kits (Tehran, IR Iran).

### 3.5. Ultrasonography of the Liver

We used U.S for all cases after laboratory tests. For 213 patients in the same condition, U.S was performed. (By 3.5-MHz Probe, Logiq 200 PRO, Tokyo, Japan). To avoid inter-operator discordance, we used an expert radiologist for all evaluations. An expert radiologist performed all U.S evaluations for 213 patients and repeated the suspicious ultrasonographies. During eleven weeks, U.S was performed for two-hundred and thirteen patients. All results of U.S were divided into mild, moderate, and severe stages under the supervision of an expert radiologist. After the final review of the U.S results, the data entered the statistical analysis phase.

### 3.6. Statistical Analysis

All risk factors and grades of ultrasonography were compared with the values obtained using one way ANOVA. An ordinal logistic regression model was used to estimate the probability of ultrasonography grade. Ordinal logistic regression is a type of logistic regression (link) which deals with ordinal dependent variables (in this case U.S grades were divided into mild, moderate, and severe stages). Regarding the stages, there are multiple response levels and they have a specific order, but no exact spacing exists between the levels of U.S grades, therefore ordinal logistic regression analysis was used to predict U.S in patients with independent variables such as AST, ALT, Fasting Blood Glucose (FBS), cholesterol, and TG. The Link function is a complementary log-log “log (−log (1−x))” used for estimation of the model. Pearson chi-square was used, and odds ratio were calculated with Crosstabs procedure. The Statistical Package for the Social Science software (SPSS, version 16.0) was used. P values < 0.05 were considered as statistically significant.

### 3.7. Definitions and Normal Ranges

**AST (aspartate aminotransferase): **is an enzyme found in liver and other organs like heart and muscle cells. It rises in liver diseases and stress condition. Its abnormal level is ≥ 33 U/L (30).

**ALT (alanine aminotransferase):** is a liver enzyme and rises in hepatic injury. Its abnormal level is > 55 IU/L (33).

**TG (Triglyceride):** TG is carried in chylomicrons and very low-density lipoprotein particles, and its level is measured in fasting state. More than 200 mg/dL is abnormal (34).

**Cholesterol:** is a fat-like substance, presents in cell membranes and steroid hormones, and is transported by lipoproteins in the blood. More than 200 mg/dL is abnormal (35).

**FBS (Fasting Blood Glucose):** used to screen diabetes mellitus and glucose tolerance. Its normal range is < 100 mg/dL (36, 37).

**Body Mass Index (BMI):** is an index calculated by dividing a person's weight in kilograms by the square of their height in meters. BMI provides a reliable indicator of body fatness for most people, and is used to screen weight categories that may lead to health problems. People are classified as normal weight (BMI < 25.0 kg/m^2^), overweight (BMI ≥ 25.0 and ≤ 29.9 kg /m^2^), and obese (BMI ≥ 30.0 kg /m^2^).

## 4. Results

Two-hundred and thirteen cases including 140 male (65.7 %) and 73 female (34.3 %) aged 16 to 64 years, were studied. Overall, 60 cases were obese (28.2 %), and 94 cases were overweight (44.1 %). Most patients in our research were 31 to 40 years old, who were most in the grade 2 of U.S. A comparison of data by ANOVA (post-hoc tests) in different USG showed an association between TG, BMI, age and FBS with USG, but only BMI (P ≤ 0.001) and TG (P = 0.011) were statistically significant to USG. There were no significant values regarding age, AST, ALT, cholesterol, and glucose levels. The most important clinical characteristics of patients with NAFLD are described in Table 1. The odds ratio (OR) for independent variables associated to U.S grades "mild" and "moderate to severe" are presented in Table 2. 

**Table 1.** Demographical, Clinical, and Serological Data According to ANOVA Analysis of Patients With NAFLD

Grade	%	Min	Max	P value
**Age**	-	-	-	0.502
1	36	17	60	-
2	48	22	60	-
3	31	16	64	-
Total	100	16	64	-
**BMI ** ^**a**^	-	-	-	< 0.001
1	35	18	38	-
2	48	22	37	-
3	17	26	35	-
Total	100	18	38	-
**AST ** ^**a**^	-	-	-	0.154
1	36	15	181	-
2	49	16	291	-
3	15	17	196	-
Total	100	15	291	-
**ALT ** ^**a**^	-	-	-	0.521
1	36	17	519	-
2	48	14	512	-
3	16	20	208	-
Total	100	14	519	-
**FBS ** ^**a**^	-	-	-	0.169
1	36	65	136	-
2	49	20	299	-
3	15	76	241	-
Total	100	20	299	-
**TG ** ^**a**^	-	-	-	0.011
1	31	80	350	-
2	53	51	908	-
3	16	63	900	-
Total	100	51	908	-
**CHOL ** ^**a**^	-	-	-	0.141
1	31	80	362	-
2	52	80	425	-
3	17	80	267	-
Total	100	80	425	-

^a^ Abbreviations: ALT: alanine aminotransferase; AST: aspartate aminotransferase; BMI: body mass index; CHOL: cholesterol; FBS: fasting blood glucose; TG: triglyceride.

**Table 2.** Odds Ratio (OR) for Independent Variables Associated to Ultrasonography Grades “Mild” and “Moderate” to “Severe”

Variables	OR	95% CI	P value
**Gender**	1.34	0.64-2.86	0.28
**AST ** ^**a**^	1.48	0.83-2.66	0.11
**ALT ** ^**a**^	1.22	0.68-2.18	0.29
**TG ** ^**a**^	2.62	1.19-5.76	0.01
**CHOL ** ^**a**^	1.94	0.88-4.24	0.06
**FBS ** ^**a**^	1.83	0.79-4.25	0.10

^a^ Abbreviations: ALT: alanine aminotransferase; AST: aspartate aminotransferase; CHOL: cholesterol; FBS: fasting blood glucose; TG: triglyceride.

**Figure 2.**
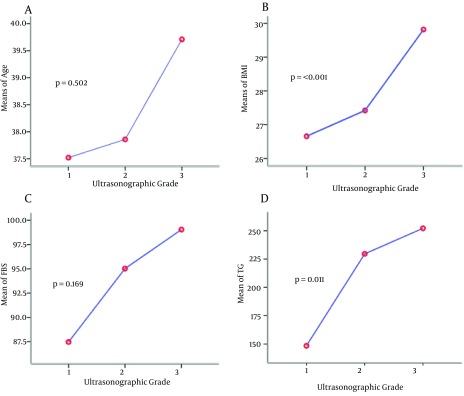
Raw Data Plots of Single Selected Variables vs. Grade of Liver Disease. (A) Age vs. Grade. (B) Body Mass Index (BMI) vs. Grade. (C) Fasting Blood Sugar (FBS) vs. Grade. (D) Triglycerides (TG) vs. Grade

The ANOVA comparisons of mean liver function tests including ALT and AST levels in different USG are shown in Figure 2. There was no statistically significant difference between mean ALT and AST levels when the moderate and severe groups were compared (P = 0.41, 0.21, respectively). Relation of each variable with USG was plotted. Gradient of changes about age and BMI between grades 2 to 3 and about TG and FBS between grades 1 to 2 show more correlation. Ordinal logistic regression used to determine the most effective predictors on USG showed that BMI and AST were the most associated factors to predict fatty liver severity according to the grade of ultrasonography in patients (Table 2). 

## 5. Discussion

Today, early diagnosis of NAFLD is an important goal, especially in overweight people, because this disease is associated with severe liver disorder (8, 38). In this study we evaluated BMI and laboratory parameters and compared data to find associations between these parameters and the grade of fatty liver in U.S. NAFLD is divided into primary and secondary types (18). Primary type is common among overweight and obese people , diabetes mellitus (type 2), and metabolic syndrome. We investigated cases with primary type of fatty liver. Subgroup analysis in this study showed that age, BMI, FBS and TG were related to USG, and severity of fatty liver but only BMI and TG had significant correlation with NAFLD severity and high grade ultrasonography (by ANOVA) Table 1. 

The effect of BMI is similar to the results of Rocha and Fassio, and we suggested that BMI measurement is helpful for evaluation of NAFLD (14, 39). Also in Ordinal logistic regression, BMI was predictor for USG. As well as some explained articles, BMI is predictor of NAFLD severity or significantly higher in the patients with fatty liver (Tables 2 and 3) (26, 40, 41). AST had a prediction role in the severity of disease and U.S grade. However, it has not been a reliable finding, because AST level changed in many conditions such as systemic disorder (Tables 2 and 3) (28, 30). Nevertheless, in several papers like Purnak T, Sogabe M and Lin YC, AST was a predictor of NAFLD severity and U.S significantly (26, 27, 29).

**Table 3.** Ordinal Logistic Regression

Variable	Estimate	Std. Error	Wald	Sig
**BMI ^a^**	- 0.076 ^b^	0.098	0.605	0.437
**Age**	+ 0.096 ^b^	0.051	3.553	0.059
**AST ** ^**a**^	- 0.005	0.007	0.455	0.500
**ALT ** ^**a**^	+ 0.000	0.005	0.003	0.957
**FBS ** ^**a**^	+ 0.006	0.005	1.631	0.202
**TG ** ^**a**^	+ 0.001	0.001	0.340	0.560
**CHOL ** ^**a**^	+ 0.002	0.002	0.746	0.388

^a^ Abbreviations: ALT: alanine aminotransferase; AST: aspartate aminotransferase; CHOL: cholesterol; FBS: fasting blood glucose; TG: triglyceride.

^b^ In this table estimated amount represents the importance of variables in the prediction model. The negative sign shows positive effect, and positive sign shows negative effect.

On the other hand, we could not find any significant association between ALT and the grade of U.S that is consistent with many researches (26, 29, 42, 43), but Kennedy showed that ALT is not specific for the diagnosis of NAFLD. This could be due to high level of malnutrition among people of Ilam, with low levels of Pyridoxine intake in routine diet. Also, Rafeey stated that total cholesterol, ALT and AST were correlated with the severity of NAFLD at U.S grading, but it is not about FBS and TG. In our study ALT, cholesterol and FBS had no significant correlation with USG (44). About TG, older studies showed correlation with the severity of fatty liver disease at U.S and high levels of TG, like our study, USG were higher (Tables 1 and 2) (31, 42, 43) and the total cholesterol did not have significant association with NAFLD and USG similar to the results of Thomopoulos KC and Nakhjavani M (45, 46). It seems that inappropriate food habits, indiscriminate uses of fat and physical inactivity are the reasons for this dyslipidemia, which could be improved or treated by changing lifestyle and diet among high-risk people, especially in early stages.

### 5.1. Strengths and Limitations

An informed consent was obtained from all patients who took part in the study after explaining the study goals. Most important defects of ultrasonography are, overlap between close grads because ultrasonography is a visual rating system, and highly operator-dependent (47). U.S diagnosis of NAFLD in people but in obesity (BMI > 30) and morbid obesity (BMI > 35), specificity of it decreasing and negative predictive value increased. To obtain more and better data, we used an expert radiologist and repeated suspicious ultrasonographies. Biopsy does not affect the treatment course, and according to the Sleisenger and Fordtran's Gastrointestinal and Liver Disease Text book, for defining NAFLD, liver biopsy is controversial and not necessary, so we did not perform liver biopsy. The golden standard to diagnose NAFLD is liver biopsy, but it not necessary to all patients or mild form of disease (14, 48). Many studies during the recent years indicated that liver enzymes including AST and ALT can be helpful to detect or predict NAFLD and its grading, but due to the high variability of liver enzymes, it does not seem that these enzymes have a definite role in the accurate diagnosis of fatty liver. Mikako Obika also showed that liver enzymes do not appear to have any association with diagnose of NAFLD (49). On the other hand, association of lipid profiles, especially triglycerides, and fatty liver and NAFLD appears firmer and more logical. Hence, we recommend to take lipid profiles into account while evaluating fatty liver and NAFLD. Of course, for confirmation of this association, more comprehensive studies are needed. Finally, in early stage of NAFLD, clinical or laboratory tests are not sensitive, and we suggested simultaneous use of a triple method including taking a complete history and physical examination (especially BMI), laboratory findings (lipid profile, especially TG), and ultrasonography.
